# A critique of the US Surgeon General’s conclusions regarding e-cigarette use among youth and young adults in the United States of America

**DOI:** 10.1186/s12954-017-0187-5

**Published:** 2017-09-06

**Authors:** Riccardo Polosa, Christopher Russell, Joel Nitzkin, Konstantinos E. Farsalinos

**Affiliations:** 1 Centro Prevenzione e Cura del Tabagismo, Azienda Ospedaliero Universitaria “Policlinico-V. Emanuele”, Catania, Italy; 2 Dipartimento di Medicina Clinica e Sperimentale, Università di Catania, Catania, Italy; 3 UOC di Medicina Interna e d’Urgenza, Azienda Ospedaliero Universitaria “Policlinico-V. Emanuele”, Catania, Italy; 4 Centre for Substance Use Research, Glasgow, UK; 5 R Street Institute, Washington D.C., USA; 6 Department of Cardiology, Onassis Cardiac Surgery Center, Sygrou 356, 17674 Kallithea, Greece; 7 Department of Pharmacy, University of Patras, 17674 Rio, Greece

**Keywords:** US Surgeon General, E-cigarettes, Nicotine, Youth, Adolescents

## Abstract

**Background:**

In December 2016, the Surgeon General published a report that concluded e-cigarette use among youth and young adults is becoming a major public health concern in the United States of America.

**Methods:**

Re-analysis of key data sources on nicotine toxicity and prevalence of youth use of e-cigarettes cited in the Surgeon General report as the basis for its conclusions.

**Results:**

Multiple years of nationally representative surveys indicate the majority of e-cigarette use among US youth is either infrequent or experimental, and negligible among never-smoking youth. The majority of the very small proportion of US youth who use e-cigarettes on a regular basis, consume nicotine-free products. The sharpest declines in US youth smoking rates have occurred as e-cigarettes have become increasingly available. Most of the evidence presented in the Surgeon General’s discussion of nicotine harm is not applicable to e-cigarette use, because it relies almost exclusively on exposure to nicotine in the cigarette smoke and not to nicotine present in e-cigarette aerosol emissions. Moreover, the referenced literature describes effects in adults, not youth, and in animal models that have little relevance to real-world e-cigarette use by youth. The Surgeon General’s report is an excellent reference document for the adverse outcomes due to nicotine in combination with several other toxicants present in tobacco smoke, but fails to address the risks of nicotine decoupled from tobacco smoke constituents. The report exaggerates the toxicity of propylene glycol (PG) and vegetable glycerin (VG) by focusing on experimental conditions that do not reflect use in the real-world and provides little discussion of emerging evidence that e-cigarettes may significantly reduce harm to smokers who have completely switched.

**Conclusions:**

The U.S. Surgeon General’s claim that e-cigarette use among U.S. youth and young adults is an emerging public health concern does not appear to be supported by the best available evidence on the health risks of nicotine use and population survey data on prevalence of frequent e-cigarette use. Nonetheless, patterns of e-cigarettes use in youth must be constantly monitored for early detection of significant changes. The next US Surgeon General should consider the possibility that future generations of young Americans will be less likely to start smoking tobacco because of, not in spite of, the availability of e-cigarettes.

## Background

As ‘America’s doctor,’ the US Surgeon General on the Public Health Service is the most authoritative voice on matters of public health, preventive medicine, and health promotion in the United States of America. A core responsibility of the Surgeon General is to communicate the best available scientific information to the public regarding ways to improve personal and public health. The landmark 1964 report of the Surgeon General on the adverse health consequences of tobacco smoking [1], for example, heightened global awareness about the harms of smoking and inspired public health and legislative initiatives to reduce the burden of tobacco use on individuals who smoke, their families, and wider society. Subsequent Surgeon General’s reports have expanded our understanding of the health consequences from active and passive smoking across an expanding list of diseases and organ systems [2, 3]. The Surgeon General’s reports have been instrumental in communicating to both smokers and healthcare professionals that quitting smoking fully and at the soonest opportunity is the best action a smoker can take to improve his or her health in the short- to long-term, and consequently, steady progress in curtailing the rate of smoking-related morbidity and mortality in the USA has typically followed the publication of these reports.

On 8 December 2016, now former US Surgeon General, Vivek M. Murthy published his first report on e-cigarette use among youth and young adults in the USA [4]. Based on a review of data on the patterns, perceptions, correlates, and health effects of e-cigarette use among youth and young adults in the USA, the Surgeon General concluded that exposure to nicotine and other chemicals through e-cigarettes pose serious health risks to young people (chapter 3), and that e-cigarettes are being used by a rapidly increasing proportion of youth and young people in the USA (chapter 2). As such, the Surgeon General states in the report’s preface that “e-cigarette use among U.S. youth and young adults is now a major public health concern.”

Obviously, efforts should be made to deter use of e-cigarettes by unintended (from a public health perspective) population subgroups, specifically non-smokers and youth. However, since e-cigarettes could be part of a broader strategy for reducing tobacco-related harm in the population, policy decisions that would change the availability and attractiveness of e-cigarettes should weigh the likely protective and harmful effects of e-cigarette use by different population groups. In this paper, we present data that show e-cigarette use to be substantially less harmful and less frequent among young people in the USA than the Surgeon General suggests. In particular, we examine data that were available to, but not included or minimally discussed by, the Surgeon General, that suggest the risks of long-term inhalation of e-cigarette vapor is likely to carry a fraction of the risk associated with long-term inhalation of cigarette smoke, and that majority of e-cigarette use among US youth is infrequent and experimental, minimal among never-smoking youth, and does not actually involve nicotine use. Together, we argue that these data do not support the claim that e-cigarette use among US youth is of substantial public health concern, and instead argue e-cigarettes may represent a significant opportunity to accelerate the USA’ progress towards its first generation of smoke-free Americans.

## Critique of chapter 2: patterns of e-cigarette use among US youth and young adults

### Foreword

Chapter 2 of the Surgeon General’s report describes patterns of e-cigarette use among youth (12–17 years) and young adults (18–24 years). The report makes repeated statements about ‘the number of young people who use e-cigarettes’; for example, “E-cigarette use has increased considerably in recent years, growing an astounding 900% among high school students from 2011 to 2015” (p. 7). This statement is true when ‘use’ is measured as the proportion of young people who have *ever* tried using an e-cigarette, even once, in their lifetime. However, one-time or experimental use of an e-cigarette is extremely unlikely to increase any individual’s risk for developing any disease known to be caused by smoking. Rather, pre- and post-market population models of the public health impact of e-cigarette use are more meaningfully informed by data on the prevalence of three characteristics of e-cigarette use that were afforded only limited discussion by the Surgeon General: frequency of e-cigarette use, the nicotine concentration of e-cigarettes used, and the smoking status of the e-cigarette user.

### Ever-use vs. frequent use

The Surgeon General’s report draws heavily from the *National Youth Tobacco Survey* (NYTS) and the *Monitoring the Future Study* (MTF) [5–8], two nationally representative surveys of US youth. Despite being reported by these surveys, the Surgeon General’s report provides little discussion of the rate of frequent e-cigarette use, defined as use on at least 20 of the past 30 days, among US youth. Measures of e-cigarette use, which do not capture frequency, intensity, or reasons for use, are largely uninformative and provide misleading conclusions about the individual as well as public health impact of e-vapor products [9].

While these surveys do indeed show youth rates of ever-use of an e-cigarette have increased by several hundred per-cent in recent years, they also show youth rates of frequent use of an e-cigarette, which is more strongly indicative of a behavior likely to be sustained, and so, more strongly associated with health outcomes, have remained very low between 2011 and 2015. Data from the 2015 NYTS, for example, reveal that, among middle school students, 13.5% have ever used an e-cigarette (i.e., ever-users) and 5.3% have used an e-cigarette at least once in the past 30 days, but only 0.6% have used an e-cigarette on at least 20 of the past 30 days (i.e., frequent users) [4] (p. 29). Among high school students, the respective rates were 37.7, 16.0, and 2.5% [4] (p. 30). Similar patterns have been observed among young adults too, with the 2013–2014 National Adult Tobacco Survey finding rates of ever-use, current use, and frequent use among those aged 18–24 years to be 35.8, 13.6, and 2.0%, respectively [4] (p. 38). The 2014 MTF survey, too, revealed that although past 30-day e-cigarette use was reported by 17.2% of 12th graders, only 6.6% had used e-cigarettes for > 5 days in the past month [10].

### Frequent e-cigarette use according to smoking status

The 2014 MTF survey showed that frequent e-cigarette use was extremely rare among never-smoking youth: only 1.7 and 0.7% of never smokers were using e-cigarettes for > 5 days and 20–30 days of the past month, respectively [7]. In contrast, 14.7% of current regular smokers and 15.0% of youth who smoked regularly in the past were currently using an e-cigarette frequently. A secondary analysis of the 2014 NYTS data showed that 87% of past 30-day e-cigarette users had ever used a tobacco product while 63% reported using a tobacco product in the past 30 days [11]. However, less than 0.1% of never-users of tobacco had used e-cigarettes for > 10 days of the past month [11]. Similarly very low rates of frequent e-cigarette use among never-smoking US youth have also been observed among youth in several other countries [12, 13]. For example, although approximately 12% of adolescents in the UK reported ever-use of e-cigarettes, only 0.7–1% reported use of an e-cigarette more than once weekly, with most being smokers [14, 15].

### Nicotine-containing e-cigarette use

Next, the Surgeon General states that “…most e-cigarettes contain nicotine, which can cause addiction and can harm the developing adolescent brain.” [1] (p. 7). Consumption of nicotine via e-cigarette aerosol is an important determinant of an individual’s risk for dependence on e-cigarettes and progression to frequent use or transition to smoking tobacco cigarettes. However, while most e-cigarettes may indeed contain nicotine, data suggest the majority of ever-user US youth use e-cigarettes that do not contain nicotine. Miech et al. recently reported data from the 2015 MTF survey showing that 65–66% of e-cigarette ever-user students in the 8th, 10th, and 12th grade had, at last use, used an e-cigarette that did not contain nicotine (i.e., only contained flavorings); only 13–22% of ever-user students were using nicotine-containing e-cigarettes [16]. For current e-cigarette users, 59–63% reported using e-cigarettes containing ‘just flavorings’ at last use. Similar findings have been observed in Canada, where 70% of high school e-cigarette ever-users had never used an e-cigarette that contained nicotine [17]. Thus, the rate of nicotine-containing e-cigarette use appears to be a small proportion of overall e-cigarette use in this population.

### Rate of youth e-cigarette use over time

The alarm raised by the Surgeon General comes at a time when prevalence of ever-use of an e-cigarette among US youth appears to be stabilizing, even declining. The MTF survey found no change in prevalence of past 30-day use between 2014 and 2015, despite the fact that, in 2015, the survey questions were broadened to say “electronic vaporizers such as e-cigarettes” instead of just “e-cigarettes”. While the 2015 NYTS reported an increase in past 30-day use from 2014 (9.3%) to 2015 (11.3%) the rate of increase has significantly declined compared to the change from 2013 (3.1%) to 2014. Recently, data from the 2016 MTF survey [18] revealed a substantial decline in e-cigarette use compared to 2015. Specifically, the 2016 prevalence of past 30-day use was 6.2% in 8th graders (from 9.5% in 2015), 11.0% in 10th graders (from 14% in 2015), and 12.5% in 12th graders (from 16.0% in 2015). Similar to 2015, the majority of adolescents were using e-cigarettes that did not nicotine.

### Youth e-cigarette use and smoking rates

The greatest public health concern about e-cigarettes, however, is not the rate at which youth are currently using e-cigarettes, but the rate at which youth use of e-cigarettes may increase rates of youth use of more harmful tobacco products (e.g., starting to smoke cigarettes). Setting aside momentarily the evidence that the vast majority of youth e-cigarette use in the USA is found among youth who are already smoking, the rate at which non-smoking youth who use e-cigarettes may become smokers as a consequence of having used e-cigarettes is a legitimate concern and a pressing public health policy question.

Broadly, there are two trajectories in which a youth’s likelihood of initiating smoking varies as a function of his/her e-cigarette use, and two trajectories in which the likelihood of initiating smoking occurs independently of e-cigarette use. In the worst-case scenario for public health, e-cigarette use may (for example, by habituating a young non-smoker to the effects of nicotine and the sights, smell, and feel of inhaling and exhaling a visible vapor) increase interest in smoking cigarettes among youths who were unlikely to have started smoking had they not started using e-cigarettes first (i.e., a putative causal effect), resulting in net harm to the youth population. In the best-case scenario for public health, use of an e-cigarette (particularly those that do not physically resemble a conventional cigarette and those containing flavors that are not available through conventional cigarettes) may provide a sufficiently pleasurable experience that discourages initiation of cigarette smoking among youth who were more likely to have started smoking cigarettes had they not started using e-cigarettes first (i.e., a putative protective effect), resulting in net avoided harm to the youth population. Then there are two trajectories in which cigarette smoking and e-cigarette use are unrelated: youth who are likely to start smoking *even if they do not start using e-cigarettes*, and youth who are unlikely to start smoking *even if they do start using e-cigarettes*. Studies that report data on the prevalence of these four mutually exclusive relationships between e-cigarette use and smoking initiation will provide a good basis for estimating the net health impact of e-cigarettes on US youth. Thus, while the low rates of current use and frequent use of an e-cigarette use among US youth revealed by multi-year cross-sectional surveys do not depict e-cigarette use among US youth as a major public health concern at present, monitoring for evidence of an increased use of e-cigarettes by non-smoking youth, and an increased rate of smoking initiation among the small proportion of non-smoking youth who use e-cigarettes is essential.

Determining the rates at which e-cigarette use prevents, causes, and coincides with smoking initiation among youth requires longitudinal studies to follow young people over time, ideally over several years, to adequately characterize the rates of youth smoking initiation associated with prior regular, experimental, one-time, and no use of an e-cigarette. These studies should ideally also seek to identify the combinations of e-cigarette device formats, flavors, nicotine strengths, and use settings most strongly associated with youths’ increased and reduced odds for future smoking initiation.

Five longitudinal studies cited in the Surgeon General report claimed that e-cigarette use at baseline predicted smoking at follow-up [19–23]. However, all studies suffered from the issues discussed above. They only assessed ever-use [19–22] or past 30-day e-cigarette use [23] and did not assess the nicotine content of e-cigarettes used. There was no evidence that adolescents were regular e-cigarette users at baseline, and no evidence that they were smoking cigarettes regularly at follow-up. These aspects are crucial in supporting a gateway hypothesis, i.e., that adolescents became addicted to e-cigarettes and then transition to addiction to cigarette smoking. Moreover, it is not clear how e-cigarettes could be causally linked to cigarette smoking, unless adolescents became addicted to nicotine and/or the act and rituals of inhalation through e-cigarette use and then were curious to try smoking cigarettes. It is possible that these adolescents may have become smokers even in the absence of e-cigarettes; in that case, initiation of e-cigarette use may be related to easier access or cheaper price of first-generation disposable products and a predisposition of these subjects to engage in an inhalational habit. Thus, the ‘gateway hypothesis’ can be neither supported nor rejected by the findings of these studies. In contrast, two studies found that the implementation of restrictions on e-cigarette sales to adolescents was associated with an increased smoking rate among adolescents [24, 25] and another study reported an association between restrictions on e-cigarette sales an increased smoking rate among pregnant youth and young adults [26]. Although the evidence is not conclusive, the potential for e-cigarettes to have a primary prevention role, and the potential for restrictions on e-cigarettes sales to increase smoking initiation among US youth, should be carefully considered. Continuous monitoring of youth transitions between cigarette smoking and e-cigarette use through prospective cohort studies are a public health imperative, but such studies should assess as their primary dependent variable the prevalence of regular or frequent e-cigarette and cigarette smoking at baseline and follow-up, respectively.

It should also be emphasized that the increasing rate of ever-use of e-cigarettes among US youth has coincided with the sharpest declines in youth smoking rates in many decades. Data from the NYTSs show past 30-day smoking prevalence in high school students decreased from 15.8% (2011) to 12.7% (2013) to 9.2% (2014), while in 2015 no further decrease in prevalence was observed. The 2015 MTF survey showed a continuous decline in past 30-day smoking prevalence to 7.0% in 2015 compared to 11.7% in 2007. In 2016, further declines were observed in all school grades (12th grade = 10.5%; 10th grade = 4.9%; 8th grade = 2.6%) [27].

Among young adults too, between 2010 and 2015, the period in which the Surgeon General points to rapidly increasing use of e-cigarettes among US young adults, the prevalence of smoking reduced by 54% among 18–19 year-old males and by 64% among 18–19 year-old females. These reductions are three times and five times larger, respectively, than the reductions observed between 2005 and 2010, when e-cigarette use was essentially zero. Given the cross-sectional design of the MTF, NYTS, and NATS, no conclusive determination can be made of the role played by e-cigarettes in the observed incremental declines in smoking prevalence among US youth and young adults between 2010 and 2015. This possibility certainly exists, though is not addressed in the Surgeon General’s report. At the very least, available data appear reassuring that e-cigarettes are not decelerating let alone reversing declining rates of youth smoking.

### Use of flavored e-cigarettes

The Surgeon General report presents data from population surveys that indicate most adolescents who have ever used an e-cigarette have used flavored e-cigarettes. Again, however, the important question is not what flavors are being used, but with what effect are different flavors being used by youth, young adults, and adults? It should be expected that good flavors will attract consumers of all ages. Flavors appear to play an important role in perceived satisfaction and self-reported effectiveness of e-cigarettes among adults who have used e-cigarettes to stop smoking. A survey of adult e-cigarette users, most of whom were former smokers, indicated that flavors played an important role in their efforts to reduce or quit smoking with the use of e-cigarettes [28]. Most participants were using multiple flavors on a regular basis. Additionally, adult smokers appear to prefer tobacco-flavor when they start using e-cigarette, but as e-cigarette use develops, preferences appear to dimish for tobacco flavor and grow for sweet and fruit flavors [28, 29]. The use of these non-tobacco flavors may help suppress craving for cigarettes and so help the e-cigarette user to sustain abstinence from smoking, since such flavors should be less likely than tobacco flavor to cue smoking as a conditioned response. Other surveys have also shown that a small minority of adult e-cigarette users are using flavorless liquids [30]. Thus, the decision to ban or restrict flavors should depend on the balance between the health benefits to adults who manage to reduce or quit smoking by switching to use of flavored e-cigarettes, and the need to protect youth who likely would have never smoked, if it is shown that flavors are indeed a significant determinant of regular e-cigarette use and subsequent smoking initiation.

## Critique of chapter 3: health effects of e-cigarette use among US youth and young adults

### Foreword

Chapter 3 gives an overview of the scientific literature on the health effects attributable to e-cigarette use, including reports of harmful consequences attributable to e-cigarettes battery explosions and fires, as well as to accidental overdose of nicotine-containing e-liquids. The Surgeon General’s document is clear that there are no existing youth-related health outcomes of exposure to nicotine in e-cigarette aerosol emissions, and the evidence is limited to studies of adults and/or experimental models (i.e., animal and in vitro data). With regard to potential harmful effects of nicotine exposure in utero and during adolescence, chapter 3 draws from existing literature on exposure to cigarette smoke because no data are available for exposure to nicotine carried in e-cigarette aerosol emissions. By equating findings obtained from smoking conventional cigarettes smoking to vaping e-cigarettes, a comparable health risk of e-cigarette use by youth is ultimately portrayed.

### Effects of nicotine inhalation by the e-cigarette user

Nicotine uptake through e-cigarette use depends on the nicotine concentration (mg/ml) of the e-liquid being consumed, the design of the device, the user’s puffing behavior, and the user’s experience with the product [31–34]. This section of the report speculates that high-performance e-cigarettes may promote higher absorption of nicotine than tobacco cigarettes. To date, there is no evidence of this ever occurring; no evidence of nicotine overdosing has been reported, even under conditions of compensatory puffing. Levels of plasma nicotine and cotinine (a stable metabolite of nicotine) in vapers are comparable to that in cigarette smokers [35, 36].

The causal link between cigarette smoking and cardiovascular disease is undisputed [2, 3]. The evidence that nicotine might be a risk factor for the development of cardiovascular disease is far less compelling. It has been suggested that nicotine may contribute to atherogenesis directly through activation of nicotinic acetylcholine receptors in the blood vessels [37, 38] and indirectly via formation of inflammatory mediators with pro-atherosclerotic activity [39]. This evidence is drawn from laboratory studies using higher doses and more prolonged exposures than would ever be seen in real life. However, current evidence suggests that, at concentrations observed in smokers, nicotine has a minor effect on the initiation or propagation of atherosclerosis [40]. It seems obvious that the toxic mixture of polycyclic hydrocarbons, tobacco-specific nitrosamines, oxidizing agents, carbon monoxide, and thousands of other chemicals in the cigarette smoke is responsible for most, if not all of the atherogenesis, not the nicotine [41]. In his discussion of short-term clinical trials of e-cigarettes in adults, the Surgeon General fails to acknowledge the impacts on blood pressure (BP), heart rate (HR) and aortic stiffness are transient [42–46], and therefore unlikely to cause clinically relevant harm. We know that the cardiovascular risk of nicotine pharmaceuticals and snus is much lower than the risk from smoking [47–49]. Hypertensive smokers who switch to e-cigarettes show decreased systolic and diastolic blood pressure and improved blood pressure control [50].

The relevance of the Surgeon General’s discussion of nicotine carried by e-cigarette aerosol is questionable because it relies almost exclusively on exposure to nicotine carried by cigarette smoke. The referenced literature describes effects in adults and animal models that have little relevance to real-world human e-cigarette use. The animal models feature high nicotine dose, excessive duration of continuous exposure, and subcutaneous or oral administration.

Furthermore, the evidence that smoking by adolescents and pregnant women might lead to impaired cognition, attention deficit disorders and mood disorders later in life, is inconclusive. The literature on this topic includes several studies showing that anxiety disorders may precede tobacco use and dependence among adolescents [51]. These bidirectional associations between adolescent smoking and anxiety in early adulthood do not prove causality. The same applies to other disorders, such as ADHD [52] and depression [53]. In any case, these studies do not show that any increased risk of cognitive impairments and mood disorders can be attributed to nicotine as distinct from other constituents of tobacco smoke. Other important confounders such as genetic predisposition and social influences may also underlie the development of these disorders [54–56]. Lastly, a recent Cochrane review suggests that use of medicinal nicotine by pregnant women who smoke has no negative effects on birth outcomes [57]. A randomized trial of use of nicotine during pregnancy showed that children born to smokers who used pharmaceutical nicotine products during pregnancy were more likely to have better developmental outcomes than children of smokers who received a placebo [58]. All of these findings support the premise that the adverse outcomes are most likely due to other toxicants carried in tobacco smoke, not to the nicotine.

Thus, the Surgeon General’s report does not provide convincing evidence that nicotine decoupled from the by-products of combusted tobacco causes actual harm to infants born to NRT-using mothers, adolescents, pregnant women, or women of reproductive age. Although obviously not recommended for non-smoking adolescents and pregnant women to use any nicotine-containing products recreationally, the role of lower-risk nicotine products may be valuable as a smoking reduction or cessation aid. Although e-cigarettes are not completely risk-free, a review of the evidence commissioned by Public Health England (PHE) stated that the harm associated with e-cigarettes “is likely to be extremely low, and certainly much lower than smoking” [59]. Most importantly, in relation to pregnancy, e-cigarette does not emit carbon monoxide [60], which is particularly harmful to developing babies. That said, the risks to a fetus from e-cigarette vapor exposure are unknown. Pregnant women who smoke should be advised to access behavioral support and, if needed, licensed nicotine replacement therapy (NRT) products to help them quit smoking. However, if they choose to use an e-cigarette, and e-cigarette help them to quit smoking and stay smoke-free, significant harm will be prevented to both the mother and unborn baby compared to continuing to smoke [61].

### Effects of the inhalation of constituents other than nicotine

Smokers smoke for the nicotine, but die from the tar in tobacco smoke [62]. E-cigarettes do not contain tobacco leaf, do not combust any other organic material, and so do not produce smoke. The Surgeon General acknowledges that e-cigarettes carry far fewer toxins than cigarettes, and so are likely far less hazardous to the user’s health. For example, e-cigarette users who do not smoke show far lower urine levels of tobacco smoke toxicants than cigarette smokers [63–65]. This section of the report places excessive emphasis on potential absolute risks of e-cigarette use and fails to consider the likelihood that substituting e-cigarettes for conventional cigarettes can prevent much if not all of the harm caused by smoking. It references methodologically flawed analytical laboratory, animal, and cell studies, which have been largely misinterpreted [60, 66, 67].

The report exaggerates the toxicity of propylene glycol (PG) exposure in human and animal studies, as reviewed previously [60] and highlights findings of eye irritation, cough, and airway obstruction [68, 69] without noting that these irritant effects quickly disappear [70, 71]. Characteristically, Weislander et al. reported a small but significant decrease of FEV1/FVC (*P* = 0.049) [68]. This was partly driven by an increase in FVC (denominator), which is paradoxical and not observed in any disease condition [72]. Moreover, the report does not consider the possibility that PG might have health benefits. PG, in its aerosol form, is a potent bactericidal agent [73]. Regular exposure may contribute to the prevention of respiratory tract infections and exacerbations of chronic obstructive pulmonary disease (COPD) [74, 75]. Additionally, the remarkable fall of exhaled carbon monoxide from highly toxic levels in smokers to within normal limits in e-cigarette users [63, 76–78] is not mentioned in the report.

### Effects of toxicants produced during aerosolization

Thermal degradation of propylene glycol and vegetable glycerin has the potential to form low molecular weight carbonyls, including formaldehyde, acetaldehyde, and acrolein [79]. Under conditions of realistic use, these toxicants are found at far lower levels in e-cigarette aerosol than in cigarette smoke, and below levels known to cause substantial harm to humans [80]. Overheating e-liquids can generate levels of carbonyls exceeding those found in cigarette smoke [80–82]. This overheating, however, is extremely unlikely to occur in normal use because of the extremely unpleasant taste known as the “dry puff phenomenon” [80]. Newer e-cigarette models feature automatic temperature control to prevent overheating. These and future technological advances together with the implementation of quality and safety standards promise to further reduce the already very low levels of carbonyls in e-cigarette aerosol. Indeed, the totality of the best available evidence of the toxicity and carcinogenicity of cigarette smoke and e-cigarette aerosol suggests that while the effects of long-term aerosol inhalation are as yet unknown, the risk posed by long-term inhalation of aerosol produced by properly manufactured e-cigarettes is unlikely to exceed 5% of the risk associated with long-term inhalation of cigarette smoke [83].

## Conclusions

Optimal public health policy would minimize consumption of combustible cigarettes and minimize long-term use of any other nicotine delivery product among youth and pregnant women. If cigarettes did not exist, e-cigarettes would present public health harms, but no likely benefits. However, in the presence of cigarettes as the dominant nicotine delivery product in Western society, e-cigarettes offer lower risk to users, and may reduce long-term nicotine use if proven to be less addictive and easier to quit than cigarettes.

Closer inspection of data reported by the two nationally representative surveys of US youth upon which the Surgeon General based the claim that ‘e-cigarettes are a threat to public health’ show the majority of e-cigarette use among US youth is infrequent and experimental, and minimal among never-smoking youth. Additionally, the majority of the very small proportion of US youth who do use an e-cigarette frequently are actually using e-cigarettes that do not contain nicotine. The Surgeon General dedicates limited or no discussion to these data. Lastly, the increasing prevalence of e-cigarette use between 2010 and 2015 has coincided with the sharpest declines in the smoking rate among US youth and young adults on record.

Compared to conventional cigarettes, e-cigarettes substantially reduce the user’s exposure to harmful and potentially harmful constituents of tobacco smoke, and may have a reduced liability for abuse. E-cigarettes differ from other non-tobacco nicotine products in their greater acceptance and popularity among adult smokers. The Surgeon General should also consider the possibility that e-cigarettes may have the potential to reduce the likelihood of smoking initiation among youth who may be especially at risk for initiating smoking in the absence of e-cigarettes. National and local monitoring patterns of e-cigarette and cigarette use among youth should be continuous, with emphasis of measurement on the prevalence of regular or frequent use, and the prevalence of likely harm-increasing and harm-reducing transitions between e-cigarette use and smoking prevalence. Ultimately, regulatory decisions that affect the availability and attractiveness of e-cigarettes should be determined by the likely net population impact of taking versus not taking a particular course of action, which includes the impact on youth and adult smokers.
